# Risk factors and clinical outcomes of incomplete endoscopic resection of small rectal neuroendocrine tumors in southern China: a 9-year data analysis

**DOI:** 10.1093/gastro/goac084

**Published:** 2022-12-30

**Authors:** Xiaoduan Zhuang, Shaoheng Zhang, Guiquan Chen, Zongqi Luo, Huiqin Hu, Wenfeng Huang, Yu Guo, Yongwen Ouyang, Liang Peng, Qing Qing, Huiting Chen, Bingsheng Li, Jie Chen, Xinying Wang

**Affiliations:** Department of Gastroenterology, Zhujiang Hospital, Southern Medical University, Guangzhou, P. R. China; Department of Gastroenterology, Zhujiang Hospital, Southern Medical University, Guangzhou, P. R. China; Department of Gastroenterology, Affiliated Dongguan Hospital, Southern Medical University (Dongguan People's Hospital), Dongguan, P. R. China; Department of Gastroenterology, Affiliated Dongguan Hospital, Southern Medical University (Dongguan People's Hospital), Dongguan, P. R. China; Department of Gastroenterology, Huizhou First Hospital, Huizhou, P. R. China; Department of Gastroenterology, Huizhou First Hospital, Huizhou, P. R. China; Department of Gastroenterology, The First Affiliated Hospital, Sun Yat-sen University, Guangzhou, P. R. China; Department of Digestive Diseases, The First People's Hospital of Foshan, Foshan, P. R. China; Department of Gastroenterology, The First Affiliated Hospital of Guangzhou Medical University, Guangzhou Medical University, Guangzhou, P. R. China; Department of Gastroenterology, The Third Affiliated Hospital of Guangzhou Medical University, Guangzhou, P. R. China; Department of Gastroenterology and Hepatology, Guangzhou Digestive Disease Center, Guangzhou First People's Hospital, School of Medicine, South China University of Technology, Guangzhou, P. R. China; Department of Gastroenterology, Huizhou First Hospital, Huizhou, P. R. China; Center for Neuroendocrine Tumors, Fudan University Shanghai Cancer Center, Shanghai, P. R. China; Department of Head & Neck Tumors and Neuroendocrine Tumors, Fudan University Shanghai Cancer Center, Shanghai, P. R. China; Department of Gastroenterology, Zhujiang Hospital, Southern Medical University, Guangzhou, P. R. China

**Keywords:** rectal neuroendocrine tumors, incomplete resection, risk factors, clinical outcomes

## Abstract

**Background:**

The histologically complete resection (CR) rate of small rectal neuroendocrine tumors (RNETs) is unsatisfactory at the first endoscopy. Risk factors and clinical outcomes associated with incomplete resection (IR) have not been explicitly elucidated. This study aims to explore the relevant factors of IR.

**Methods:**

This retrospective study reviewed patients with small RNETs (≤10 mm) in eight centers from January 2013 to December 2021. Clinicopathological characteristics and clinical outcomes were compared between the CR and IR groups, and the polypectomy and advanced treatment groups.

**Results:**

Of the 326 patients included, 83 (25.5%) were diagnosed with IR. Polypectomy (odds ratio [OR]* *=* *16.86), a central depression (OR = 7.50), and treatment in the early period (OR = 2.60) were closely associated with IR. Further analysis revealed that an atypical hyperemic appearance (OR = 7.49) and treatment in the early period (OR = 2.54) were significantly associated with the inappropriate use of polypectomy (both *P *<* *0.05). In addition, a total of 265 (81.3%) were followed up with a median follow-up period of 30.9 months. No death, metastasis, or recurrence was found during the follow-up period.

**Conclusions:**

Polypectomy, a central depression, and treatment in the early period were risk factors for IR. Further, an atypical hyperemic appearance and treatment in the early period were significant predisposing factors for inappropriate choice of polypectomy. For histologically incompletely resected small RNETs, follow-up may be a safe and feasible alternative to rigorous salvage therapy.

## Introduction

Neuroendocrine tumors (NETs) are the most frequent endocrine tumors of the gastrointestinal tract [1, 2] with the rectum as the third most common site [3]. The incidence of rectal NETs (RNETs) has steadily and remarkably increased over the past several decades, reportedly detected in 0.05%–0.07% of patients undergoing screening endoscopy [4]. Endoscopic mucosal resection (EMR) and endoscopic submucosal dissection (ESD) are widely used for the resection of small RNETs (≤10 mm in size) without risk factors for metastases [5]. Cold-forceps polypectomy and snare polypectomy are not recommended due to the lack of assurance of sufficient CR of the lesion margins. Even for larger RNETs (10–19 mm) without metastasis and muscularis propria invasion, ESD is effective for resection of RNETs due to the advantages of en bloc resection and accurate pathological assessment [6, 7]. However, despite the rapid development of endoscopic technologies, it remains difficult in clinics to achieve a satisfactory complete resection (CR) rate for RNETs. The overall endoscopic CR rate was reportedly as low as 39% [8]. So far, some studies have explored risk factors associated with incomplete resection (IR) [8–12]. However, most were conducted in single centers with relatively small sample sizes and conflicting results.

To date, there has been no consensus on a consolidated therapeutic strategy for incompletely resected RNETs. The necessity for salvage treatment for small RNETs resected incompletely remains uncertain. Even different guidelines have different recommendations [13–15]. The main reason for the controversies is the uncertainty of the prognosis of lesions resected incompletely. In clinical practice, a large proportion of patients with IR lesions refused salvage treatment for fear of salvage-therapy-related complications and economic burden. It has been reported that 72.7% (56 of 77) of those with incompletely resected small RNETs refused salvage treatment [16]. For such small IR lesions without additional therapy, it was still unclear whether regular follow-up is safe and feasible.

Therefore, the aim of this multicenter retrospective study of a large cohort was to identify risk factors and clinical outcomes associated with IR of small RNETs, especially those removed by polypectomy.

## Materials and methods

### Study approval

The study protocol was approved by the Institutional Review Committees of all participating hospitals and conducted in accordance with the Ethical Principles for Medical Research Involving Human Subjects as defined in the Declaration of Helsinki. Ethical approval was obtained from the Ethics Committee of Zhujiang Hospital, Southern Medical University (approval number: 2021-KY-070–01).

### Study cohort

The medical records of patients who underwent endoscopic resection of RNETs in eight hospitals (Zhujiang Hospital, Dongguan People's Hospital, Huizhou First Hospital, The First Affiliated Hospital of Sun Yat-sen University, The First People's Hospital of Foshan, The First Affiliated Hospital of Guangzhou Medical University, The Third Affiliated Hospital of Guangzhou Medical University, and Guangzhou First People's Hospital) located in Guangdong province, P. R. China from January 2013 to December 2021 were retrospectively reviewed. In this study, the early period was from January 2011 to December 2016, while the late period was from January 2017 to December 2021. The key words, including “rectal neuroendocrine tumor” and “rectal carcinoid,” were used for search patients diagnosed with RNETs after endoscopic resection in the endoscopic pathology reporting system of different centers. Subsequently, the above initially included patients with RNETs were further screened according to inclusion and exclusion criteria through the clinical medical records system. Patients who met the following criteria were initially included in this study: (i) NETs diagnosed histologically, (ii) NETs located in the rectum, and (iii) RNETs resected via endoscopy. The exclusion criteria were as follows: (i) tumor size of >10 mm, (ii) existing distant or lymph node metastasis, (iii) concomitant colonic cancer or synchronous gastrointestinal NETs, and (iv) incomplete medical records, especially clinicopathologic and endoscopic findings.

### Endoscopic findings and treatment methods

**Figure 1.**
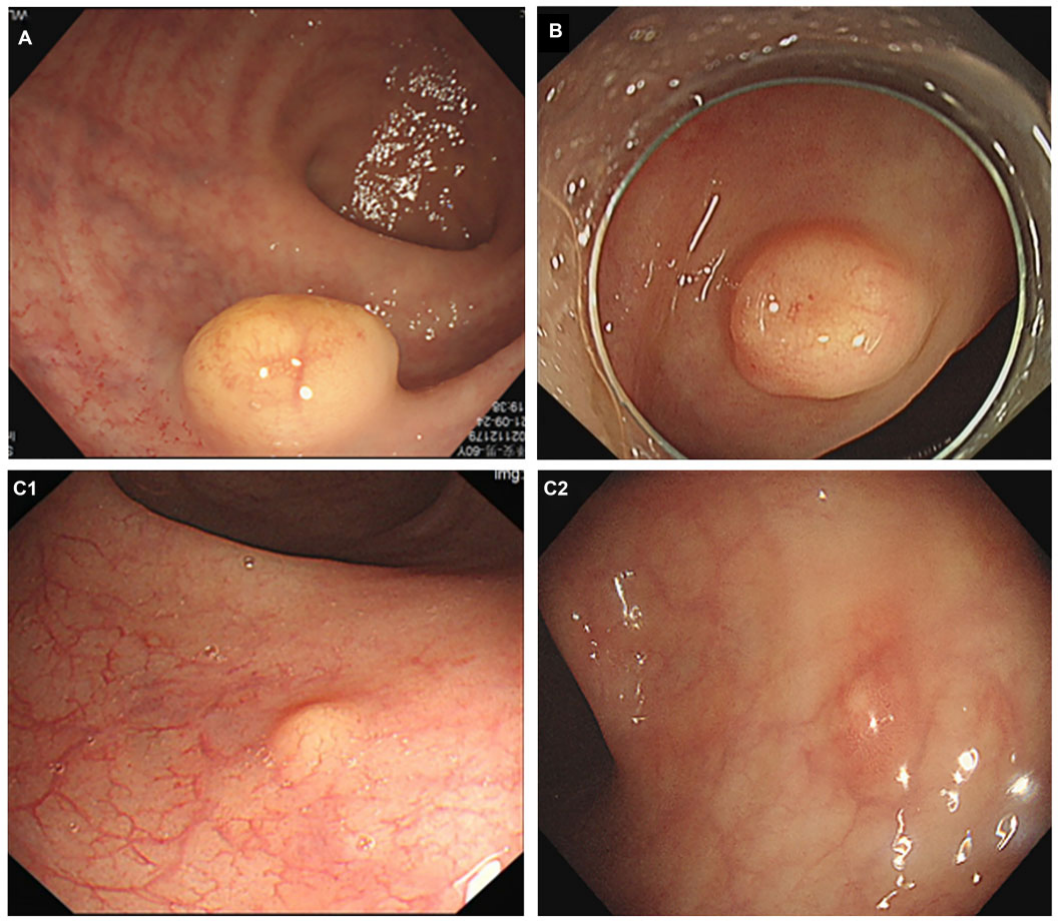
Different colors and morphologies of rectal neuroendocrine tumors (RNETs) under endoscopy. (A) The tumor was classified as a protruded type with a yellowish white surface color. (B) The tumor was defined as the depression type and it was covered in a yellowish white surface. (C) The tumors belonged to the slightly elevated type: (C1) the surface color was yellowish white; (C2) the surface color was hyperemic.

Endoscopic features, including tumor location, size, morphology, and surface color, were collected. As shown in Figure 1, tumor morphology during the first endoscopy was divided into three types. Protruding lesions were classified as pedunculated (Ip), semi-pedunculated (Isp), or sessile (Is), while those slightly elevated corresponded to type IIa and IIb lesions. Regardless of the presence of a protrusion or slight elevation, RNETs with a depressed surface were classified as the central depressed type.

The endoscopic intervention methods for RNETs, which included the use of cold-forceps polypectomy, cold-snare polypectomy, EMR, and ESD, were assessed and documented for each case. Simple endoscopic treatment was defined as the use of forceps polypectomy or snare polypectomy, while advanced endoscopic treatment was defined as the use of EMR or ESD. Procedure-related complications, which mainly included bleeding and perforation, were also collected from clinical records.

### Histopathologic assessments

All lesions were assessed independently by experienced pathologists at each center. The lesion margin was classified as positive, negative, or indeterminate. CR (R0) was defined as en bloc resection with no residual tumor cells in the lateral and vertical margins, while “indeterminate” was defined as the inability to assess the margins because of fragmentation or electrocautery artifacts. In this study, indeterminate and positive margins were both classified as IR. The grade of each RNET was determined in accordance with the 2019 World Health Organization (WHO) criteria [17].

### Follow-up

Patients with R1 resection but refusing salvage treatment were followed with colonoscopy and abdominal computed tomography (CT) at 6 months after endoscopic resection. Thereafter, colonoscopy and abdominal CT were performed annually. For patients with R0 resection, annual colonoscopy and abdominal CT were recommended. Follow-up data were retrospectively collected from medical records. Recurrence was defined as local recurrence, lymph node metastasis, or distant metastasis during the follow-up period. Local recurrence was defined as the detection of a RNET at the primary site after 1 year or after one negative follow-up endoscopy.

### Statistical analyses

Categorical variables are presented as frequencies and continuous variables as the mean ± standard deviation. Univariate analysis was performed using the *χ*^2^ test or Student’s *t*-test. Multivariable logistic regression was performed to identify associations between pathological outcomes and the collected factors. The Kaplan–Meier method was used to estimate local recurrence-free survival. A probability (*P*) value of <0.05 was considered statistically significant. All statistical analyses were performed using IBM SPSS Statistics for Windows, version 22.0 (IBM Corporation, Armonk, NY, USA).

## Results

### Patient and tumor characteristics

Of the 388 patients with primary RNETs initially identified, 62 were excluded, which included 32 with tumors of >10 mm, 2 with concomitant colonic cancer, 5 with lymph node metastasis as determined by preoperative endoscopic ultrasonography (EUS), 6 with synchronous gastrointestinal NETs, and 17 with incomplete clinicopathological records. Thus, 326 patients (208 males and 118 females) were finally included in the study.

As shown in Table 1, the median age of the patients was 47.3 (range, 17.4–76.4) years. More than 60% small RNETs were incidentally detected without any clinical symptoms. In regard to tumor characteristics, 278 (85.3%) RNETs were located in the distal rectum (<7 cm from the anal verge). The average tumor size was 0.65 cm. Only 45 (13.8%) lesions were >8 mm. Of the 326 cases, 32 (9.8%) were initially treated with cold-forceps polypectomy, 12 (3.7%) with snare polypectomy, 29 (8.9%) with conventional EMR, 6 (1.8%) with modified EMR, and 247 (75.8%) with ESD. In regard to complications, bleeding was observed in 10 (3.1%) cases and perforation in 2 (0.6%). All the above complications occurred during the ESD treatment and were eventually cured after conservative medical treatment. In regard to treatment time, 39 (12.0%) RNETs were resected in the early period and 287 (88.0%) in the late period. Resection-margin assessment indicated that 243 (74.5%) cases achieved R0 resection, 67 (20.5%) had positive vertical margins, and 3 (0.9%) had positive lateral margins, while the resection margins of the remaining 13 (4.0%) were not determined.

**Table 1.** Baseline characteristics of included patients and lesions

Variables	*N* (%)
Age, mean ± SD, years	47.3 ± 11.1
Male	208 (63.8)
Clinical symptoms	
Incidental finding	217 (66.6)
Abdominal pain/distention	71 (21.8)
Change in bowel habits	26 (8.0)
Others	12 (3.7)
Preoperative EUS performing	188 (57.7)
Distance from anal verge	
≥7 cm	48 (14.7)
<7 cm	278 (85.3)
Tumor size	
<8 mm	281 (86.2)
8–10 mm	45 (13.8)
Endoscopic intervention methods	
Cold-forceps polypectomy	32 (9.8)
Snare polypectomy	12 (3.7)
Conventional EMR	29 (8.9)
Modified EMR	6 (1.8)
ESD	247 (75.8)
Intraoperative bleeding	10 (3.1)
Perforation	2 (0.6)
Treatment time	
Early period	39 (12.0)
Later period	287 (88.0)
Tumor grade	
G1	311 (95.4)
G2	15 (4.6)
Resection margin	
R0 resection	243 (74.5)
Positive vertical margin	67 (20.5)
Positive lateral margin	3 (0.9)
Not determinable	13 (4.0)
Salvage treatment	
ESD	11 (3.4)
TEM	6 (1.8)
Surgery	1 (0.3)

SD, standard deviation; EUS, endoscopic ultrasonography; ESD, endoscopic submucosal dissection; EMR, endoscopic mucosal resection; G, grade; TEM, transanal endoscopic microsurgery.

Of the above 83 lesions resected incompletely under endoscopy, 18 lesions (21.7%) received salvage treatment, including 11 lesions treated by ESD, 6 lesions by transanal endoscopic microsurgery (TEM), and 1 lesion by surgery. However, 8 (44.4%) lesions were found to have no residual tumor cells in post-operative pathology. The remaining 65 (78.3%) patients refused salvage treatment.

### Clinicopathological factors related to IR

The overall IR rate in this study was 25.5% (83 of 326). As shown in Table 2, there was no significant difference in the median age, male-to-female ratio, tumor size, or tumor location between the CR and IR groups (all *P *>* *0.05). However, significantly more lesions in the IR group had central depressions as compared with the CR group (16.9% vs 5.8%, *P *=* *0.002).

**Table 2.** Univariate analysis of the related factors of incomplete resection

Variables	Incomplete resection group (*n *=* *83)	Complete resection group (*n *=* *243)	*P*-value
Patient characteristics			
Age, mean ± SD, years	47.0 ± 10.7	47.4 ± 11.3	0.818
Male	51 (61.4)	157 (64.6)	0.605
Endoscopic features			
Central depression			0.002
Present	14 (16.9)	14 (5.8)	
Absent	69 (83.1)	229 (94.2)	
Tumor size			0.349
<8 mm	69 (83.1)	212 (87.2)	
8–10 mm	14 (16.9)	31 (12.8)	
Distance from anal verge			0.175
≥7 cm	16 (19.3)	32 (13.2)	
<7 cm	67 (80.7)	211 (86.8)	
Treatment-related factor			
Preoperative EUS performing	30 (36.1)	158 (65.0)	<0.001
Endoscopic treatment			<0.001
Polypectomy^a^	35 (42.2)	9 (3.7)	
EMR	9 (10.8)	26 (10.7)	
ESD	39 (47.0)	208 (85.6)	
Intraoperative complication	1 (1.2)	11 (4.5)	0.308^b^
Treatment time			0.002
Early period	18 (21.7)	21 (8.6)	
Later period	65 (78.3)	222 (91.4)	
Work experience			0.507
<10 years	39 (47.0)	104 (42.8)	
≥10 years	44 (53.0)	139 (57.2)	
Pathological features			
Tumor grade			0.544^b^
G1	78 (94.0)	233 (95.9)	
G2	5 (6.0)	10 (4.1)	
Mitotic rate			0.269^b^
<2/10 HPF	81 (97.6)	241 (99.2)	
≥2/10 HPF	2 (2.4)	2 (0.9)	
Lymphovascular invasion	2 (2.4)	0 (0)	0.064^b^
CgA staining positive	38 (45.8)	95 (39.1)	0.284
Syn staining positive	82 (98.8)	239 (98.4)	1.000^b^

SD, standard deviation; EUS, endoscopic ultrasonography; ESD, endoscopic submucosal dissection; EMR, endoscopic mucosal resection; HPF, high power field; CgA, chromogranin A; Syn, synaptophysin.

a Containing cold-forceps polypectomy and snare polypectomy.

b *P*-value is calculated by using Fisher exact test.

Significantly fewer RNETs in the IR group were assessed by EUS preoperatively as compared to the CR group (36.1% vs 65.0%, *P *<* *0.001). As the first endoscopic treatment, 42.2% of the RNETs in the IR group were resected with the use of polypectomy. Not surprisingly, significantly fewer RNETs were resected with the use of ESD in the IR group than the CR group (47.0% vs 85.6%, *P *<* *0.001). There were no significant differences in the incidences of intraoperative bleeding and perforation between the two groups (*P *>* *0.05). Notably, RNETs treated in the early period were more likely to be classified as IR than CR (21.7% vs 8.6%, *P *=* *0.002). However, the percentage of cases treated by experienced endoscopists (work experience of ≥10 years) showed no significant difference between the two groups (53.0% vs 57.2%, *P *=* *0.507). According to histological assessments, there were no statistical differences in tumor grade, mitotic rate, lymphovascular invasion, and immunohistochemical staining for chromogranin A (CgA) and synaptophysin (Syn) between the two groups (all *P *>* *0.05).

Work experience, which is considered to be closely related to CR in clinical practice, was also included in the multivariate analysis. Further multivariate logistic regression analysis demonstrated that a central depression vs absence (odds ratio [OR]* *=* *7.50, *P *<* *0.001), employing polypectomy treatment vs ESD and EMR (OR = 16.86, *P *<* *0.001), and treatment in the early period vs the late period (OR = 2.60, *P *=* *0.031) were independent risk factors for IR (Table 3). Both preoperative EUS assessment and work experience were not independent predictors of IR (both *P *>* *0.05).

**Table 3.** Multivariate logistic regression analysis of factors predictive of incomplete resection

Factor	Status	OR	95% CI	*P-*value
Central depression	Present	7.50	(3.095, 18.144)	<0.001
EUS assessment	Yes	0.56	(0.294, 1.054)	0.072
Endoscopic treatment	Polypectomy	16.86	(6.958, 40.865)	<0.001
Work experience	<10 years	1.08	(0.569, 2.045)	0.816
Treatment time	Early period	2.60	(1.094, 6.196)	0.031

CI, confidence interval; OR, odds ratio; EUS, endoscopic ultrasonography; ESD, endoscopic submucosal dissection.

### Factors associated with improper use of polypectomy treatment at first endoscopy

As described above, improper use of polypectomy was the greatest risk factor for IR. Hence, factors influencing the endoscopist to adopt inappropriate use of polypectomy as the first endoscopic treatment were compared between the polypectomy and advanced endoscopic treatment group. As shown in Table 4, univariate analysis indicated that there were no significant differences in age, sex, tumor size, or tumor location between the two groups (all *P *>* *0.05). However, protrusion or slight elevation of the lesion, a hyperemic surface, work experience of the endoscopist, and the treatment period were significantly associated with the use of polypectomy (all *P *<* *0.05). Furthermore, multivariate analysis revealed that only a hyperemic appearance vs yellowish white color (OR = 7.49, *P *=* *0.001) and treatment in the early period vs the later period (OR = 2.54, *P *=* *0.033) were independent risk factors for the use of improper polypectomy as the initial treatment strategy (Table 5).

**Table 4.** Factors associated with employing polypectomy treatment strategy at first endoscopy

Variables	Polypectomy treatment group (*n *=* *44)	Advanced treatment group^a^ (*n *=* *282)	*P*-value
Age, mean ± SD, years	46.3 ± 10.8	47.4 ± 11.2	0.532
Male	24 (54.5)	184 (65.2)	0.169
Morphology			0.025^b^
Protruding	10 (22.7)	24 (8.5)	
Slightly elevated	32 (72.7)	232 (82.3)	
Centrally depressed	2 (4.5)	26 (9.2)	
Lesion color			<0.001
Yellowish white	37 (84.1)	276 (97.9)	
Hyperemic	7 (15.9)	6 (2.1)	
Tumor size			0.330
<8 mm	40 (90.9)	241 (85.5)	
8–10 mm	4 (9.1)	41 (14.5)	
Distance from anal verge			0.827
≥7 cm	6 (13.6)	42 (14.9)	
<7 cm	38 (86.4)	240 (85.1)	
Endoscopist’s work experience			0.029
<10 years	26 (59.1)	117 (41.5)	
≥10 years	18 (40.9)	165 (58.5)	
Treatment time			0.004
Early period	11 (25.0)	28 (9.9)	
Late period	33 (75.0)	254 (90.1)	

SD, standard deviation.

a Including lesions resected by using endoscopic mucosal resection or endoscopic submucosal dissection.

b *P*-value is calculated by using Fisher exact test.

**Table 5.** Multivariate logistic regression analysis of factors about inappropriate choice of polypectomy treatment strategies

Factor	Status	OR	95% CI	*P*-value
Lesion color	Hyperemic	7.49	(2.267, 24.764)	0.001
Morphology	Protruding or centrally depressed	1.69	(0.761, 3.744)	0.197
Work experience	<10 years	1.86	(0.926, 3.719)	0.081
Treatment time	Early period	2.54	(1.080, 5.955)	0.033

CI, confidence interval; OR, odds ratio.

### Follow-up

Of the 326 patients, 265 (81.3%) were followed up in this study. The median follow-up period was 30.9 (mean, 25.3 ± 19.4; range, 4.1–120.2) months. No patient died during the follow-up period. Of the 243 patients in the CR group, 173 (71.2%) received follow-up and showed no recurrence or metastasis. Of the 83 patients in the IR group, all 18 (21.7%) patients who received salvage treatment underwent follow-up colonoscopy and abdominal CT. No patients found recurrence or metastasis over the median follow-up period of 23.5 (mean, 27.3 ± 21.5; range, 5.8–77.9) months. Of the remaining 65 patients in the IR group who refused salvage treatment, 45 patients (69.2%) underwent follow-up colonoscopy and abdominal CT with a median follow-up period of 25.7 (mean, 27.4 ± 24.1; range, 4.1–120.2) months. Surprisingly, no local recurrence or metastasis was found in those patients either during the follow-up period.

## Discussion

The incidence of RNETs has rapidly increased over the past few years along with the increase in screening by colonoscopy [18–20]. However, the CR rates of RNETs treated by endoscopy varied widely as reported by different studies, ranging from 39% to 85% [8, 11, 16, 21]. The overall CR rate of RNETs is reportedly only 84.08% even after the advanced endoscopic technique of ESD [22]. Though RNETs are indolent tumors, they still have metastatic potential, even of relatively small size [23]. Therefore, the analysis of IR-related factors is particularly important. Relatively few studies have explored risk factors associated with IR of RNETs and there were differences in the inclusion criteria, which resulted in conflicting conclusions [8–12]. Based on the clinicopathological characteristics of 326 patients with non-metastatic small RNETs (≤10 mm) in Southern China in the past nearly 10 years, we found that the use of polypectomy vs advanced endoscopic techniques (ESD and EMR) were significant risk factors for IR (OR = 16.86, *P *<* *0.001), consistently with two previous studies [8, 11]. Advanced endoscopic techniques, such as ESD and EMR, are clearly recommended in guidelines [24]. The CR rate in the present study was 74.5%, which was significantly higher than the 39% reported by Fine *et al.* [8], which was likely due to the greater use of ESD (75.8%) in our study.

In the present study, an atypical endoscopic morphology of a central depression was found to be another significant risk factor for IR. Depression and ulceration have been reported as the risk factors for metastasis [25]. However, previous studies of risk factors associated with IR seemed to pay little attention to the appearance of a central depression. Only two reports assessed central depression but arrived at different conclusions [9, 26]. In our study, RNETs with a central depression were closely associated with IR (OR = 7.50, *P < *0.001), possibly because this feature is associated with greater tumor size. In addition, a central depression is thought to be linked with severe interstitial reactions and fibrosis of the tumor, which could hinder CR. In addition, as the only research focusing on the treatment period, we found that treatment in the early period was correlated with a lower diagnosis rate and higher IR rate. In the early period (from January 2013 to December 2016), only 12.0% (39 of 326) of RNETs were diagnosed and treated, which included 18 (46.1%) that were classified as IR. This finding implies low awareness of the importance of screening via colonoscopy, insufficient recognition of RNETs, and lack of experience with advanced techniques by endoscopists in southern China in the early period.

As we found above, the endoscopic treatment technique is the most important risk factor for IR. Some studies have indicated that many small RNETs may be suspected of being polyps and thus removed by inappropriate simple excisional biopsy or snare polypectomy in clinics [8, 11, 27]. According to the study by Fine *et al.*, >80% of RNETs were not suspected as NETs during first endoscopy, which resulted in 74.67% (168 of 225) of RNETs being resected by polypectomy with only a 17% R0 resection rate [8]. The results indicated that the lack of recognition of RNETs seriously influences decisions of the endoscopist for treatment of RNETs. However, specific factors leading the endoscopist to have insufficient recognition of RNETs and thus to choose an inappropriate approach were not further explored in those studies. Therefore, in the present study, potentially relevant factors were further compared between the polypectomy and advanced endoscopic treatment groups, which found that an atypical hyperemic appearance and treatment time of the early period were significantly associated with the use of polypectomy. Of the 13 lesions with hyperemic appearances, 10 (76.92%) were misdiagnosed as polyps and directly removed by using polypectomy at the first endoscopic assessment, suggesting that the endoscopist should be aware of hyperemic lesions in the rectum and consider the possibility of atypical appearances of RNETs. Checking the hardness and mobility of such lesions with forceps can help to distinguish submucosal tumors from polyps, thereby facilitating appropriate treatment strategies.

To date, guidelines do not clearly indicate the necessity for salvage treatment for endoscopic IR lesions. The consensus guideline update for colorectal NETs reveals that it is unclear whether salvage therapies are really required due to limited evidence [14]. However, the review by Louis de Mestier *et al.* [28] emphasized the necessity for salvage treatment due to the potential for malignancy and progression of IR lesions. The controversies are largely due to the uncertainty as to whether the clinical outcomes of RNETs differ between CR and IR lesions. Based on the follow-up data of a large number of patients from multiple centers, we found that, whether they were CR lesions or IR lesions with or without salvage treatment, all presented excellent clinical outcomes with no disease-related deaths or recurrences. Similarly, a study conducted in South Korea of 107 patients also indicated that the clinical outcomes for RNETs (≤10 mm in size) achieved excellent outcomes after a median follow-up period of 31 months, regardless of the margin status [29]. It has been also reported that of the 428 small RNETs (<1 cm), all 54 IR cases without salvage treatment found no recurrence during the 10- to 110-month follow-up period [10]. Additionally, the research by Hyun Jung Lee *et al.* [30] showed that of 142 patients with IR lesions, ≤98 (69.0%) were willing to receive regular colonoscopy follow-up and found no recurrences and metastasis, presenting a prognosis comparable to that of the remaining 44 patients who received salvage treatment. These latest follow-up data, especially our results from multi-centers, directly questioned the necessity for rigorous salvage treatment, with strong evidence demonstrating the safety and feasibility of a regular follow-up regimen for endoscopic IR lesions. Even for IR lesions receiving salvage therapy, the positive detection rate of NET cells was not high. In the present study, <50% of IR lesions were detected to have residual RNET cells in the second pathology after salvage therapy. Another study revealed that only 10% of lesions histologically classified as IR had residual tumor cells discovered by endoscopy during the follow-up [31]. These findings suggest that positive resection margins are not always the predictors of residual tumor. The cauterization and destruction effects for the remnant tumor cells during endoscopic resection may largely explain the low detection rate of residual tumor cells in IR lesions. Salvage therapy may result in unnecessary over-treatment due to the low detection rate and favorable prognosis of residual lesions. On the other hand, there are hints that regular surveillance using radiology and colonoscopy are acceptable and safe for RNETs lesions of <10 mm to monitor the extremely low possibility of potential progress and recurrence. Larger studies with longer follow-up periods are needed to optimize endoscopic follow-up regimens.

There were some limitations to this study that should be addressed. First, although this was a retrospective study, 326 cases from eight tertiary hospitals were analysed, which largely improved the applicability and generality of the conclusions. Second, we must acknowledge that due to the relatively short median follow-up period in our study, EMR and ESD are still considered to be the preferred treatment methods for small RNETs to achieve a higher CR rate at the first endoscopic intervention. The observation of clinical outcomes may be biased due to the relatively short median follow-up period of 30.9 months. Nevertheless, there were some implications for the diagnosis, treatment, and follow-up of RNETs. Prospective studies with longer follow-up periods are warranted to confirm these findings. Third, the multidimensional comparative studies, such as economic benefits and quality of life, between IR patients who received and did not receive salvage treatment have not been explored in our study. Further studies are expected to overcome this limitation. However, our study still has important implications for clinics that regular follow-up may be a feasible alternative to rigorous salvage therapy due to the favorable clinical outcomes of IR lesions. Fourth, the clinicopathological factors were collected from different centers with inevitable differences in endoscopic recordings and histological assessments of RNETs.

## Conclusions

In summary, polypectomy treatment, RNETs with a central depression, and treatment in the early period were independent risk factors for IR, especially polypectomy treatment. Further, an atypical hyperemic appearance and treatment in the early period were significant predisposing factors for the choice of polypectomy at the first endoscopy. For small RNETs resected incompletely, regular follow-up may be a safe and feasible alternative to rigorous salvage therapy due to the favorable clinical outcomes.

## Authors’ Contributions

X.Z., S.Z., G.C., Z.L., H.H., W.H., Y.G., Y.O., L.P., Q.Q., and H.C. were involved in the acquisition and interpretation of data. X.Z. and S.Z. were involved in the analysis of data, interpretation of data, and drafting of the manuscript. B.L., J.C., and X.W. were in involved in the study design and supervision. All authors read and approved the final version of the manuscript.
